# Interventional Endoscopy for the Management of Post-Surgical Leaks and Fistulas: A Scoping Review

**DOI:** 10.3390/jcm15062291

**Published:** 2026-03-17

**Authors:** Tommaso Pessarelli, Irene Maria Bambina Bergna, Cinzia Boemo, Alberta De Monti, Marta La Milia, Cristina Marfinati Hervoso, Michela Pagliarulo, Alessandra Piagnani, Mauro Zago, Arnaldo Amato

**Affiliations:** 1 Digestive Endoscopy and Gastroenterology Department, Ospedale A. Manzoni Hospital, 23900 Lecco, Italy; t.pessarelli@asst-lecco.it (T.P.); i.bergna@asst-lecco.it (I.M.B.B.); c.boemo@asst-lecco.it (C.B.); a.demonti@asst-lecco.it (A.D.M.); m.lamilia@asst-lecco.it (M.L.M.); c.marfinatihervo@asst-lecco.it (C.M.H.); m.pagliarulo@asst-lecco.it (M.P.); a.piagnani@asst-lecco.it (A.P.); 2 Department of General Surgery, Ospedale A. Manzoni Hospital, 23900 Lecco, Italy; m.zago@asst-lecco.it

**Keywords:** post-surgical leak, post-surgical fistula, interventional endoscopy, gastrointestinal endoscopy, over-the-scope clip, endoscopic vacuum therapy, VACstent, endoscopic suturing, endoscopic internal drainage

## Abstract

**Background/Objectives:** Leaks and fistulas are serious surgical complications associated with substantial morbidity and mortality. Traditional management has relied on surgical reintervention or percutaneous drainage, both of which carry significant risks. In recent decades, interventional endoscopy has emerged as a minimally invasive alternative, offering a growing range of therapeutic options. This scoping review aimed to systematically map the available evidence on endoscopic management of post-surgical leaks and fistulas, with a focus on techniques used, indications, outcomes, and gaps in the literature. **Methods:** This scoping review was conducted according to PRISMA-ScR guidelines. PubMed/MEDLINE, Embase, and Scopus were searched from inception to 5 December 2025. Eligible studies included original studies, systematic reviews, and narrative reviews reporting therapeutic endoscopic interventions for post-surgical leaks or fistulas in any patient population. Case reports and case series with fewer than 20 patients were excluded. Data were charted on study design, surgical context, endoscopic techniques, and reported outcomes. **Results:** A total of 69 studies were included, comprising 46 original studies involving 2550 patients, along with 11 systematic reviews and 12 narrative reviews. Endoscopic techniques identified included through-the-scope and over-the-scope clipping, stenting, endoscopic vacuum therapy, internal drainage, tissue sealants, endoscopic suturing, and hybrid devices such as VAC-Stent^®^. Reported technical and clinical success rates varied widely across techniques and clinical settings, influenced by defect characteristics, timing of intervention, anatomical location, and operator experience. Endoscopic vacuum therapy was supported by the most consistent evidence, particularly for esophageal and colorectal leaks. **Conclusions:** Interventional endoscopy represents an increasingly central component in the management of post-surgical leaks and fistulas, enabling individualized, less invasive treatment strategies. However, the current evidence base remains heterogeneous and largely retrospective, underscoring the need for well-designed, multicenter prospective studies.

## 1. Introduction

### 1.1. Rationale

Post-surgical leaks and fistulas are among the most serious and potentially life-threatening complications following a wide range of abdominal, bariatric, gastrointestinal, colorectal, hepatobiliary, and thoracic surgical procedures. A leak is defined as a transmural defect that creates communication between the intra- and extraluminal compartments, whereas a fistula is defined as abnormal communication between two epithelialized surfaces [1]. These adverse events are not uncommon, with reported incidences varying according to the type of surgery, patient-related factors, and technical considerations (up to 13.1% after esophagectomy [2]). The occurrence of post-surgical leaks and fistulas is associated with substantial morbidity and mortality, prolonged hospitalization, increased healthcare costs, and impaired long-term functional and oncologic outcomes [3]. Their management is often complex and requires a multidisciplinary approach and timely intervention to prevent severe clinical outcomes. Traditional therapeutic strategies have relied on surgical reintervention or percutaneous drainage, both of which are associated with considerable morbidity [4]. Over the past two decades, interventional endoscopy has emerged as an increasingly favored option for the treatment of post-surgical leaks and fistulas. Techniques such as endoscopic stenting, clipping, internal drainage, suturing, and vacuum-assisted endoscopic therapy (EVT) have significantly expanded the therapeutic armamentarium, offering high rates of clinical success and reducing the need for reoperation in selected clinical settings [5,6]. Furthermore, endoscopic interventions offer the advantages of lower procedural morbidity, repeatability, and the ability to tailor treatment according to defect size, location, chronicity, and associated collections. Available studies differ widely in terms of patient populations, surgical indications, timing of intervention, defect characteristics, and outcome definitions [7]. Importantly, there is a lack of standardized treatment algorithms and clear, evidence-based indications regarding the optimal selection, timing, and sequencing of specific endoscopic techniques. As a result, clinical decision-making is often guided by local expertise, individual operator preference, and institutional availability rather than robust comparative evidence or consensus recommendations [8]. Given the rapidly evolving nature of these techniques and the diversity of the available data, a comprehensive mapping of the current literature is warranted.

### 1.2. Objectives

The purpose of this scoping review is to systematically explore and synthesize the available evidence on interventional endoscopy for the management of post-surgical leaks and fistulas, with the aim of identifying the techniques used, their indications, reported outcomes, and existing gaps in knowledge. This review seeks to provide clinicians with a comprehensive overview of current practices and to highlight areas where further investigation is needed. Specifically, the objective was to map the existing literature on the use of interventional endoscopy in this clinical setting.

## 2. Methods

### 2.1. Protocol and Registration

This scoping review was conducted in accordance with the PRISMA-ScR (Preferred Reporting Items for Systematic Reviews and Meta-Analyses Extension for Scoping Reviews) guidelines. The protocol was retrospectively registered on the Open Science Framework (OSF, Center for Open Science, Washington, DC, USA, https://osf.io/m3whu/overview?view_only=8484ba8fadf54f36857c6fcd272036b0, accessed on 11 January 2026).

### 2.2. Eligibility Criteria

Studies were eligible for inclusion if they reported therapeutic endoscopic interventions for the management of post-surgical leaks or fistulas. All types of surgery (e.g., gastrointestinal, bariatric, colorectal, hepatopancreatobiliary, thoracic) and all patient populations (adult and pediatric) were considered. Original studies, narrative reviews, systematic reviews, and meta-analyses published in any language were eligible. To improve the methodological robustness and generalizability of the findings, case series with fewer than 20 patients were excluded. Small case series are more prone to selection bias, center-specific effects, and imprecise outcome estimates, which may disproportionately influence qualitative synthesis and limit external validity. The ≥20-patient threshold was therefore applied to reduce small-study effects and enhance the reliability of the overall evidence synthesis. However, to describe rare or emerging techniques, information from narrative and systematic reviews was used to provide a comprehensive overview of available therapeutic options. Studies were excluded if they addressed non-surgical leaks or fistulas, described diagnostic endoscopy only, were based on animal models or ex vivo simulations, or lacked an accessible full text.

### 2.3. Search Strategy

A comprehensive literature search was conducted in PubMed/MEDLINE, Embase, and Scopus from database inception to 5 December 2025, without language or publication date restrictions. The search strategy combined controlled vocabulary terms and free-text keywords related to endoscopic therapeutic interventions (e.g., stenting, endoscopic vacuum therapy, suturing, clipping, sealants) and post-surgical gastrointestinal leaks and fistulas. Overall, the search strategy included more than 20 intervention-related and condition-related keywords combined using Boolean operators (AND/OR). The complete search strategy is reported in the Supplementary Materials [9]. Additional relevant publications were identified by screening the reference lists of included studies and gray literature sources.

### 2.4. Study Selection

**Figure 1:**
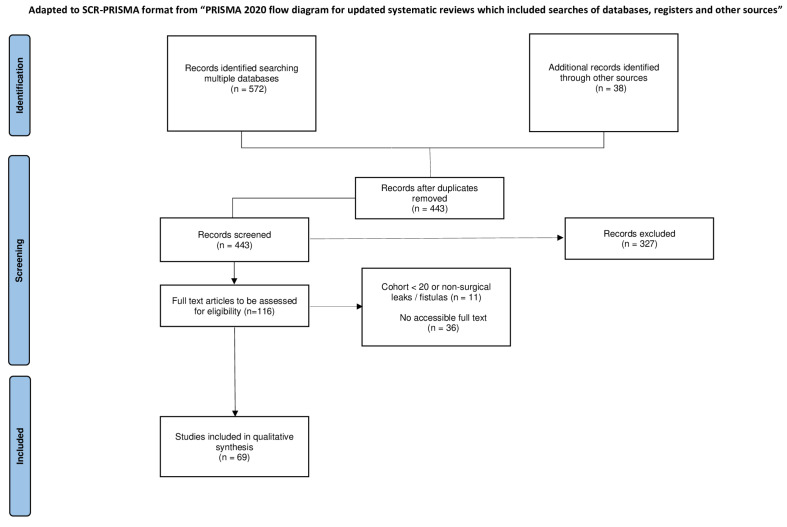
PRISMA-ScR flow diagram.

All retrieved records were imported into the Rayyan platform (Cambridge, MA, USA, https://new.rayyan.ai/reviews, last access on 6 December 2025), where duplicates were removed. Two reviewers independently screened titles and abstracts for relevance. Full-text articles of potentially eligible studies were then reviewed to confirm inclusion. Disagreements were resolved through discussion or consultation with a third reviewer. The study selection process is summarized using a PRISMA-ScR flow diagram (Figure 1).

### 2.5. Data Extraction and Synthesis

A data-charting form was jointly developed by two reviewers. Data were independently charted by both reviewers, and discrepancies were discussed and resolved through an iterative process. Extracted data included study characteristics, type of underlying surgery, nature of the post-surgical leak or fistula, endoscopic techniques and devices used, timing of intervention, technical and clinical outcomes, need for reintervention, complications, and follow-up (Supplementary Material). In accordance with recommendations for scoping reviews, no formal assessment of methodological quality or risk of bias was performed. During the preparation of this manuscript, the author(s) used generative artificial intelligence (ChatGPT, version 5.2; OpenAI, San Francisco, CA, USA) for the purposes of language review and editing and figures creation. The authors have reviewed and edited the output and take full responsibility for the content of this publication.

## 3. Results

### 3.1. Selection of Sources of Evidence

The full study selection process is presented in the PRISMA-ScR flow diagram (Figure 1).

### 3.2. Characteristics of Sources of Evidence

A total of 69 studies were included, 46 original studies (1 prospective cohort study, 30 retrospective cohort studies, 14 retrospective case series, and 1 prospective case series), comprising a total of 2550 patients, as well as 11 systematic reviews and 12 narrative reviews. The main characteristics of the included studies are summarized in Table 1 and Table 2 and the Supplementary Material.

### 3.3. Endoscopy Versus Surgery

Comparative data between endoscopic and surgical management of leaks are limited and often affected by baseline differences between treatment groups, with surgical patients typically presenting with larger or more complex defects. Nonetheless, available evidence suggests higher clinical success rates and lower mortality among patients treated endoscopically [5]. A retrospective cohort study comparing endoscopic therapy and surgery for esophageal leaks in clinically matched patients reported significantly lower mortality with EVT compared with surgery [6]. Similar findings were reported by Ascari et al. in an Italian multicenter retrospective cohort study comparing operative and non-operative management of esophageal leaks [4].

### 3.4. Through-the-Scope Clipping

#### 3.4.1. Study Selection and Characteristics

Five retrospective cohort studies [4,5,10,11,12] evaluating patients treated with through-the-scope (TTS) clips, either alone or in combination with other endoscopic therapies, were included (Table 1).

**Table 1:** Original studies including patients treated with TTS and/or OTSC alone or in combination with other therapies.

Author and Year	Design	N	Site	Technique	Technical Success	Clinical Success	Time to Closure (Days)	Need for Reintervention	Complications	Death
Ascari [4] 2024	Retrospective cohort	47	Esophagus	EVT, SEMS, TTS	NA	45.2% (first line), 87.1% (all lines)	NA	NA	NA	11.30%
Lee [5] 2013	Retrospective cohort	55	Stomach	TTS, sealants	95%	95%	NA	5%	0%	0%
Rosianu [9] 2024	Retrospective cohort	55	Esophagus	SEMS, TTS	NA	98%	NA	NA	20% (migration, perforation, hemorrhage, mediastinitis)	5%
Zhong [11] 2021	Retrospective cohort	22	Esophagus	TTS, sealants	100%	95.50%	37	NA	4.55%	4.55%
Xu [12] 2016	Retrospective cohort	28	Esophagus	TTS + NCD	NA	85.70%	NA	NA	NA	14.30%
Zhang [13] 2022	Retrospective cohort	98	Miscellaneous	OTSC	100%	55.10%	NA	NA	NA	NA
Morrell [14] 2020	Retrospective cohort	117	Miscellaneous	OTSC	NA	66.10%	NA	11.10%	NA	3.60%
Donatelli [15] 2015	Case series	30	Miscellaneous	OTSC	50%	36.60%	NA	23.30%	6.6% (migration and stricture)	NA
Mercky [16] 2015	Retrospective cohort	34	Miscellaneous	OTSC	88.20%	53%	NA	26.70%	14.7% intraprocedural	NA
Manta [17] 2015	Case series	76	Miscellaneous	SEMS, OTSC, glue, EVT	NA	80.30%	NA	19.70%	NA	1.30%
Haito-Chavez [18] 2014	Retrospective cohort	161	Miscellaneous	OTSC	93.80%	60.20%	NA	NA	NA	NA

EVT: vacuum-assisted endoscopic therapy; SEMS: self-expandable metal stents; TTS: through-the-scope; OTSC: over-the-scope clips; NCD: nasocystic drainage; NA: not available.

#### 3.4.2. Principle and Technique

TTS clips are widely available endoscopic accessories that are routinely used in clinical practice. They are available in different designs and sizes and are deployed through the working channel of the endoscope. Their effectiveness in closing chronic defects is limited by the relatively low compression force exerted on the tissue and by their tendency to spontaneously dislodge. Consequently, the presence of necrotic or inflamed tissue may result in suboptimal closure [12]. For these reasons, TTS clips are generally reserved for small leaks with regular and well-defined margins.

#### 3.4.3. Efficacy

When used in appropriately selected cases, TTS clips can achieve favorable clinical outcomes. In a retrospective cohort study, eight patients treated exclusively with TTS clips achieved a 100% clinical success rate with no reported complications, although healing times were longer compared with other endoscopic techniques [11]. Another case series reported a 95% clinical success rate for TTS clipping in gastric leaks, with or without the addition of fibrin glue [5]. Similarly, a retrospective cohort study reported an 85.7% clinical success rate when TTS clips were used in combination with nasocystic drainage (NCD) [12].

#### 3.4.4. Complications

None of the included studies reported complications directly attributable to TTS clip placement, which is consistent with the low invasiveness of this technique compared with other endoscopic interventions.

### 3.5. Over-the-Scope Clips (OTSC)

#### 3.5.1. Study Selection and Characteristics

Six studies (four retrospective cohort studies and two case series) evaluating patients with leaks and fistulas at various gastrointestinal locations treated with over-the-scope clips (OTSC), either alone or in combination with other therapies, were included (Table 1).

#### 3.5.2. Principle and Technique

OTSCs are biocompatible nitinol clips with a bear-trap design that are mounted on the tip of the endoscope, similarly to band ligation devices. Deployment is achieved by traction on a wire passed through the working channel. OTSCs provide significantly greater tissue compression compared with TTS clips [19] and are generally considered suitable for defects up to 20 mm in diameter [15]. They have been successfully used for acute gastrointestinal bleeding, closure of iatrogenic perforations, leaks and fistulas, and anchoring of self-expandable metal stents to reduce migration [20]. However, OTSC deployment requires higher technical expertise, and clip removal in the event of treatment failure may be challenging. In addition, OTSC placement may interfere with subsequent surgical procedures [15]. Adequate tissue capture may be facilitated using traction devices such as anchor graspers, twin graspers, or foreign body graspers, or by applying direct suction. Some authors have suggested abrasion of the fistula edges with a cytology brush to improve tissue adherence prior to clip deployment [21].

#### 3.5.3. Efficacy

A systematic review including patients treated with OTSCs for various indications reported a clinical success rate of 55.8% (347/622) for gastrointestinal fistulae and 72.6% (284/391) for anastomotic leaks [22], with salvage surgical intervention required in 4.7% of cases. A large multicenter retrospective cohort study evaluating the initial experience with OTSC for leaks and fistulae of the upper GI tract, midgut, and colorectum reported an overall success rate of 60.2%, which was significantly higher for leaks than for fistulae and when OTSC was used as a first-line endoscopic treatment [18]. Another case series reported a lower clinical success rate of 53% for primary closure of both upper and lower GI leaks [16]. Similarly, a case series of 30 patients with post-surgical fistulae of the upper and lower GI tract reported a technical success rate of 50% and a clinical success rate of 36%. In this study, thickened and fibrotic mucosal edges, surrounding scar tissue, and active inflammation were identified as negative predictors of OTSCs efficacy [15]. Higher success rates were reported in a meta-analysis of nine studies including 107 patients with colorectal leaks and fistulae treated with OTSCs, which demonstrated a technical success rate of 84% and a clinical success rate of 74.3% [23]. The efficacy of OTSCs may be influenced by several factors. First, operator experience appears to play a role, as data suggest that the overall success rate in high-volume centers is superior to that in low-volume centers [16]. Second, defect location is relevant; a retrospective French study demonstrated a healing rate of 88.9% for sleeve gastrectomy fistulae, which was significantly higher than the overall healing rate (*p* = 0.01) [16]. Finally, in the treatment of GI fistulae, the duration of the defect—possibly associated with increased fibrosis—seems to correlate with poorer clinical outcomes following OTSC treatment [13].

#### 3.5.4. Complications

Two of the included studies reported complication rates following OTSCs deployment. One study reported a complication rate of 6.6%, including clip migration and stricture formation [15], while another reported a 14.7% rate of intraprocedural complications [16]. In contrast, a systematic review reported that adverse events strictly related to OTSC deployment occurred in only 2.1% of cases [22]. Deformation of the lumen due to tissue retraction and failure to successfully deploy the OTSCs system because of extensive tissue fibrosis may occur in colorectal leaks and fistulae; however, these events are rare [23].

### 3.6. Endoscopic Stenting

#### 3.6.1. Study Selection and Characteristics

Our search identified 17 studies (13 retrospective cohort studies and four case series) including patients with esophageal or gastric leaks and fistulae treated with endoscopic stenting, either as monotherapy or in combination with other therapeutic modalities (Supplementary Material).

#### 3.6.2. Principle and Technique

**Figure 2:**
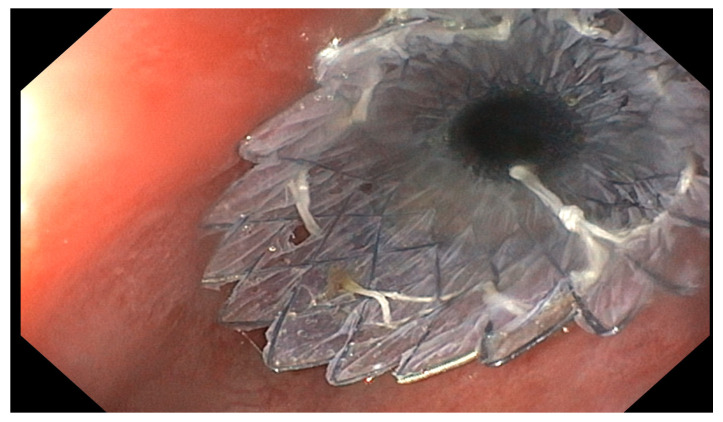
Esophageal FC-SEMS.

The rationale of endoscopic luminal stenting is to seal the defect and divert luminal contents, thereby promoting mucosal healing. Different types of stents may be used, including self-expandable plastic stents (SEPS) and self-expandable metal stents (SEMS), which can be either fully covered (FSEMS, Figure 2) or partially covered (PSEMS). SEPS are characterized by an integral polyester braid completely covered with a silicone membrane. The proximal end of the stent is flared to reduce migration and to achieve effective occlusion of esophageal leaks. Radiopaque markers at both ends and in the middle of the stent allow fluoroscopic visualization. Silicone coating prevents ingrowth and overgrowth of granulation tissue [24]. PSEMS are characterized by uncovered segments at the extremities, which may reduce migration rates but can increase the risk of tissue ingrowth and esophageal injury upon stent removal [25]. In general, PSEMS are removed 4–6 weeks after insertion, whereas FSEMS are removed after 6–8 weeks [26]. Stent length is typically selected to extend the covered portion 1–2 cm beyond the proximal and distal margins of the defect. Nasogastric tubes are not routinely placed following esophageal stent insertion [26].

#### 3.6.3. Efficacy

In a pooled analysis by van Halsema [7], 415 patients with esophageal leaks and 24 patients with fistulae treated with endoscopic stenting (plastic or metallic) achieved clinical success rates of 81.4% and 64.7%, respectively, while another meta-analysis by Schaheen reported an overall success rate of 72% [27]. A large case series demonstrated comparable clinical success rates among PSEMS, FSEMS, and SEPS (73–83%) [25]. In another case series, SEPS achieved an optimal clinical success rate (>90%) in the treatment of esophageal leaks, although concomitant interventional drainage was frequently required [24]. For SEMS, Rosianu et al. reported a clinical success rate of 98% and identified a correlation between fistula width and length of hospitalization [10]. Similarly, Freeman and colleagues reported an association between proximal esophageal injury location, defect size, and clinical failure of SEMS therapy [28]. A retrospective multicenter study, despite being limited by high mortality and low overall success rates, demonstrated that a score incorporating leak location, size, and C-reactive protein levels showed acceptable diagnostic accuracy in predicting response to endoscopic stenting for esophageal leaks. These findings suggest that alternative endoscopic approaches may be preferable in septic patients with cervical or large leaks [29]. Interestingly, a retrospective cohort study of 43 patients undergoing either EVT or stenting for esophageal leaks reported lower total mean costs for stenting compared with EVT. However, approximately 80% of total costs were attributable to intensive care unit stay, with relatively low and comparable direct costs related to endoscopic management [30]. The only data on stenting for colorectal anastomotic dehiscence identified in our search were derived from a systematic review including 68 colorectal leaks treated with stents, which reported a closure rate of 74.5% [31].

#### 3.6.4. Complications

Esophageal stenting for leaks or fistulae is commonly associated with adverse events, with some series reporting complication rates of up to 55%. Reported adverse events include dysphagia, chest pain, bleeding, perforation, hemorrhage, mediastinitis, pneumothorax, stent migration, and tissue ingrowth [10,26]. In a systematic review including 414 cases of intrathoracic leaks treated with SEPS, FSEMS, or PSEMS, stent migration was more frequent with SEPS and fully covered SEMS than with partially covered SEMS (31% and 26% vs. 12%, respectively) [27]. Anchoring the stent to the esophageal mucosa using through-the-scope (TTS) clips has been described in the literature; however, robust evidence supporting its effectiveness in preventing stent migration is currently lacking [26].

### 3.7. Endoscopic Vacuum Therapy (EVT)

#### 3.7.1. Study Selection and Characteristics

Our search identified 24 studies (one prospective cohort study, 15 retrospective cohort studies, and eight case series) including patients with esophageal, gastric, small bowel, and colorectal leaks and fistulae treated with EVT, either alone or in combination with other therapeutic approaches (Supplementary Material).

#### 3.7.2. Principle and Technique

**Figure 3:**
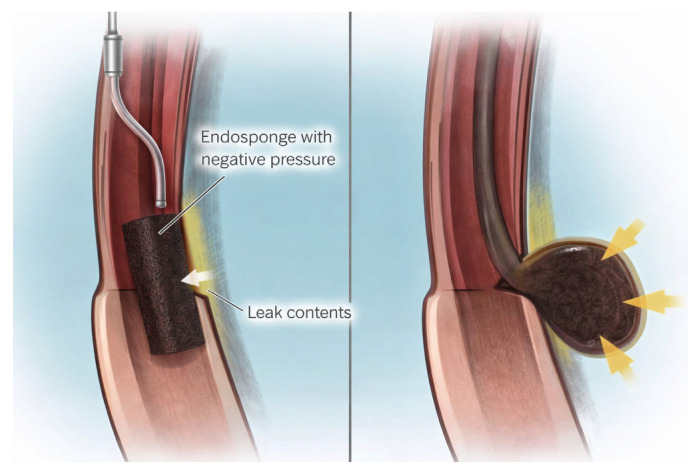
Endoluminal EVT (**left**); intracavitary EVT (**right, with arrows indicating the sponge within the abscess cavity**).

Depending on defect size, EVT is performed by endoscopic placement of an open-pore polyurethane sponge (Figure 3) either within the abscess cavity (intracavitary EVT) or within the visceral lumen to cover the leak in cases of small defects without an associated cavity (endoluminal EVT) [32]. In selected cases, intracavitary EVT may also be performed for small defects following balloon dilation of the cavity [33]. In general, however, techniques other than EVT should be preferred for very small leaks in which the tip of the endoscope cannot enter the cavity (e.g., <9 mm) [34]. For upper GI leaks, an overtube may be used to facilitate sponge insertion [33]. Subsequently, continuous negative pressure of 100–125 mmHg is applied through the drainage tube and transmitted to the tissue via the sponge, promoting mechanical cleansing of microorganisms, a reduction in interstitial edema, and stimulation of tissue granulation [35]. Sponge exchanges are typically scheduled every 3–5 days [32].

#### 3.7.3. Efficacy

Across the included studies, the clinical success of EVT ranged from 71% to 97% (Supplementary Material), with a reported technical success of 100% in all but one study, suggesting that sponge placement is technically feasible regardless of defect location. A systematic review and meta-analysis including 15 studies and 366 patients with esophageal leaks and perforations reported a pooled clinical success rate of 86.57% (95% CI, 81.94–90.61%) for leaks [36]. In a retrospective cohort study, Mennigen et al. reported a significantly higher success rate for EVT compared with FSEMS in the treatment of esophageal leaks (93.3% vs. 63.3%), with 16.6% of patients initially treated with FSEMS requiring crossover to EVT because of clinical failure [37]. Consistently, a large retrospective cohort study demonstrated higher clinical success, fewer long-term complications, and shorter treatment duration for EVT compared with SEMS in upper GI leaks [38]. One of the largest retrospective cohort studies available reported a clinical success rate of 82% for EVT. Notably, delayed initiation of EVT after leak diagnosis was the only independent factor associated with treatment failure in this study [33]. A multicenter prospective cohort study showed that EVT significantly reduced 90-day postoperative mortality and the need for esophagostomy [39]. Nevertheless, mortality rates remain substantial in some cohorts, as illustrated by a large case series reporting a mortality rate of 15.6% [35], likely reflecting the severity of post-surgical leaks, particularly in the upper GI tract. A retrospective cohort study evaluating EVT outcomes over a 10-year period demonstrated significant improvements in key clinical outcomes during the later period of EVT implementation compared with the initial phase. Specifically, time to EVT initiation decreased from 7.2 to 0.3 days, treatment duration from 25 to 14 days, and clinical efficacy improved from 80% to 91%. Additionally, a significant reduction in a composite outcome of mortality, need for patient transfer, and length of hospital stay was observed [40]. The duration of EVT and the number of treatment sessions for esophageal anastomotic leaks may be influenced by several factors, including defect size and location, operator expertise, and prior neoadjuvant therapy [41]. In a case series, Chon et al. reported a relatively short duration of EVT (mean 12 days, with a mean of two sessions) [42]. Conversely, a retrospective German cohort study comparing SEMS and EVT reported higher costs associated with EVT, resulting in approximately twice the financial deficit compared with SEMS [43]. In colorectal leaks, EVT appears to offer higher clinical success rates and better preservation of intestinal continuity compared with conventional surgical management [44] In line with this, a retrospective cohort study including 53 patients reported a clinical success rate of 93%, achieved over a median treatment duration of 21 days with a median of seven sponge exchanges. Notably, clinical success was comparable between patients with and without prior fecal diversion [45]. Restoration of bowel continuity represents a key long-term outcome in patients with colorectal anastomotic leaks. A systematic review including 827 patients treated with EVT reported an overall weighted mean rate of restored bowel continuity of 66.8%, although the analysis was limited by significant heterogeneity. Patients undergoing EVT combined with early surgical closure demonstrated higher restoration rates compared with those treated with EVT alone (82% vs. 64.7%) [46]. A German retrospective cohort study further suggested that patient-performed transanal rinsing therapy was associated with improved clinical success of EVT in colorectal leaks, although this approach requires a high level of patient compliance [47]. Compared with stenting, EVT appears to achieve higher clinical success rates, fewer complications, lower mortality, and shorter treatment duration in esophageal leaks, albeit at the cost of a higher number of endoscopic interventions [37]. These findings are supported by a systematic review reporting higher healing rates (OR 2.47), a greater number of endoscopic exchanges (pooled median difference 3.57), shorter treatment duration (pooled median difference −11.57 days), and lower stricture rates (OR 0.22) for EVT compared with stenting, with no significant differences in mortality or major treatment-related complications [48].

#### 3.7.4. Complications

According to a recent systematic review and meta-analysis, complications following EVT for esophageal leaks occur in approximately 12.6% of patients [36]. Stenosis (2–15%) and bleeding (3.1–7%) may occur in both the upper and lower GI tract, as may sponge dislocation or breakage. Rare complications include aorto-esophageal fistula, perforation, and inability to remove the sponge [33].

### 3.8. VAC-Stent

#### 3.8.1. Study Selection and Characteristics

Our search identified one prospective case series [42] including patients with esophageal leaks treated with VAC-Stent^®^. Most of the available literature consists of studies with fewer than 20 patients treated with this device.

#### 3.8.2. Principle and Technique

**Figure 4:**
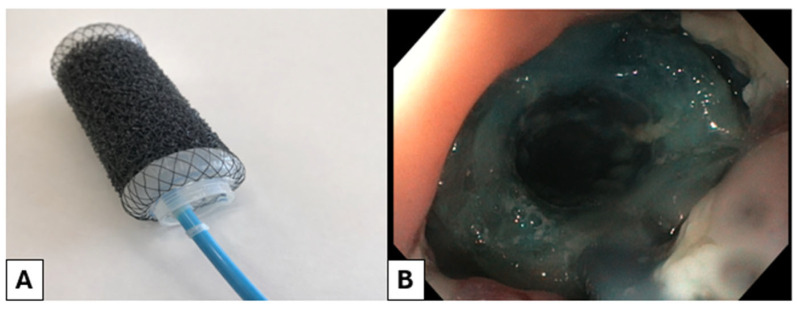
VAC-Stent ex vivo (**A**) and in vivo (esophageal leak, **B**).

The VAC-Stent^®^ (VAC StentMedtec AG, Steinhausen, Switzerland, Figure 4) is 72 mm in length, with a central diameter of 14 mm and flared ends measuring 30 mm. The device consists of a self-expandable metal stent (SEMS) covered by a 50-mm-long open-pore polyurethane foam segment in the midsection. The SEMS is constructed from nitinol wire and fully covered with a silicone-parylene layer to prevent tissue ingrowth and to seal the sponge toward the esophageal lumen. The device is deployed using a delivery system 1000 mm in length and 14 mm in diameter. The SEMS is constrained within an outer tube and mounted on an inner catheter containing a 10 F drainage tube connected to the polyurethane sponge. Deployment is achieved by retracting the outer tube from distal to proximal. The dumbbell shape of the SEMS helps prevent stent migration. Adequate coverage of the leak is achieved by positioning the polyurethane foam at least 1 cm from each flare end. Deployment is performed over a guidewire under endoscopic guidance, after which the drainage tube is connected to an electric vacuum pump applying continuous negative pressure of 65 mmHg. The VAC-Stent^®^ is typically exchanged every 3–5 days [49].

#### 3.8.3. Efficacy

A systematic review including 65 patients (70% with anastomotic leakage) reported a technical success rate of 100% and a clinical success rate of 77%. Approximately 70% of patients were able to tolerate a liquid diet during treatment [50]. In the prospective case series by Chon including 20 patients, technical success was 100%, while clinical success was 60% for the management of esophageal leaks. A potential limitation of the VAC-Stent^®^ is the delayed radial expansion of the central stent portion, possibly related to the presence of the sponge, which is relevant given the planned removal after 3–5 days [49].

#### 3.8.4. Complications

No major complications were reported. However, the risk of esophageal damage or laceration should always be considered. This risk may be mitigated by moistening the sponge and turning off the vacuum pump 2 h prior to stent removal [49]. Minor complications included sponge dislodgement, minor bleeding, and stent migration [50].

### 3.9. Internal Drainage (ID)

#### 3.9.1. Study Selection and Characteristics

Our search identified one retrospective cohort study and one case series [51,52] including patients with post–sleeve gastrectomy leaks or fistulae treated with internal drainage (ID).

#### 3.9.2. Principle and Technique

ID involves the placement of one or more double pigtail stents (DPS) to drain the abscess cavity associated with fistulae or postsurgical leaks. The rationale of ID is to internally drain any fluid collection, obstruct the leak orifice, allow early oral intake, and induce mechanical re-epithelialization of the fistula tract [8]. DPS are typically left in place for several months and removed after complete resolution of the defect. Simultaneous placement of an NCD is often performed during the first few weeks to irrigate the cavity up to three times daily, with removal guided by imaging evidence of collection regression, usually within the first month [51].

#### 3.9.3. Efficacy

In a large case series including 67 patients treated with ID for post–sleeve gastrectomy leaks, technical success was 98.5%, with a clinical success rate of 74.6% achieved over a mean of 57.5 days and 3.13 endoscopic sessions [52]. In a large retrospective cohort study, Lorenzo reported a significantly higher clinical success rate with ID compared with a “closure management” approach (endoscopic clipping, SEMS, or a combination) (86% vs. 63%, *p* = 0.043) [51].

#### 3.9.4. Complications

Stricture formation is the main complication reported, occurring in approximately 6% of cases [52].

### 3.10. Tissue Sealants

#### 3.10.1. Study Selection and Characteristics

Our search identified three retrospective cohort studies and two case series (Table 2) including patients with gastrointestinal leaks or fistulae at various sites, treated with endoscopic application of tissue sealants, either alone or in combination with other therapies.

**Table 2:** Original studies including patients treated with endoscopic application of tissue sealants, alone or in combination with other therapies.

Author and Year	Design	N	Site	Technique	Technical Success	Clinical Success	Time to Closure (Days)	Need for Reintervention	Complications	Death
Lee [5] 2013	Retrospective cohort	55	Stomach	TTS, sealants	95%	95%	NA	5%	0%	0%
Zhong [11] 2021	Retrospective cohort	22	Esophagus	TTS, sealants	100.00%	95.50%	37	NA	4.55%	4.55%
Manta [17] 2015	Case series	76	Miscellaneous	SEMS, OTSC, sealants, EVT	NA	80.30%	NA	19.70%	NA	1.30%
Lorenzo [51] 2018	Retrospective cohort	100	Stomach (sleeve)	SEMS, OTSC, NCD, sealants vs. ID	NA	86% overall, 45% OTSC, 86% ID, 63% clip/SEMS	NA	6%	50% stent migration/marginal ulcers	2%
Lippert [53] 2011	Case series	52	Miscellaneous	Sealants	NA	36.50%	70	34.60%	Abscess (46.2%), mediastinitis/peritonitis (30.8%), sepsis (28.8%), 84.6% overall	21.10%

EVT: vacuum-assisted endoscopic therapy; SEMS: self-expandable metal stents; TTS: through-the-scope; OTSC: over-the-scope clips; NCD: nasocystic drainage; ID: internal drainage; NA: not available.

#### 3.10.2. Principle and Technique

Tissue sealants have been used successfully in the management of anastomotic leaks and low-output fistulae [8]. The two most commonly used sealants are fibrin glue and cyanoacrylate. Fibrin glue, such as Tissucol Duo S (Baxter, Munich, Germany), consists of two components: one containing aprotinin and a protein concentrate derived from human plasma, and the other containing human thrombin and a calcium chloride solution. When mixed, these components form a fibrin coagulum within a short period. Fibrin glue is typically injected into the defect, after irrigation with 0.9% NaCl, via an injection catheter [53]. Cyanoacrylate (N-butyl-2-cyanoacrylate) is a synthetic adhesive that polymerizes upon contact with moisture, promoting tissue healing. It possesses strong adhesive, resistant, and antibacterial properties, making it suitable for use in the gastrointestinal tract and infected areas [8].

#### 3.10.3. Efficacy

In a large German monocentric case series, Lippert reported a clinical success rate of 36.5% when fibrin glue (median 13.9 mL over four sessions) was used alone and 55.6% when combined with other endoscopic treatments [53], with a median treatment duration of 70 days. Local application of smaller volumes of fibrin glue (2–4 mL), with or without concomitant clipping, yielded optimal clinical results in small leaks after gastric surgery [5]. A retrospective cohort study also reported use of fibrinogen, bovine thrombin, and aprotinin as tissue sealants for esophageal leaks, achieving a clinical success rate of 92.4% [12].

#### 3.10.4. Complications

Reported complications, including abscess formation, mediastinitis or peritonitis, and sepsis, appear to be more closely associated with unsuccessful treatment rather than direct adverse effects of endoscopic tissue sealant application.

### 3.11. Endoscopic Suturing

#### 3.11.1. Study Selection and Characteristics

Our search identified three retrospective cohort studies [54,55,56] including patients with gastrointestinal leaks or fistulae at various locations treated with endoscopic suturing, either alone (two studies) or in combination with stenting.

#### 3.11.2. Principle and Technique

Endoscopic suturing is a technique that allows a single operator to perform surgical-quality full-thickness closure of GI wall defects using a flexible endoscope [54]. Among available platforms, the OverStitch system (Apollo Endosurgery, Austin, TX, USA) has become the main endoscopic suturing device, with the latest version compatible with most currently used endoscopes. Endoscopic suturing requires operator expertise and specific training, limiting its use primarily to tertiary centers. Suturing can be particularly challenging in narrow luminal spaces or when the defect site is tangential. Additionally, as with surgical sutures, successful closure of large defects requires robust and healthy tissue margins [8].

#### 3.11.3. Efficacy

Across the included studies, technical success ranged from 95.8% to 100%, while clinical success ranged from 55.6% to 80% (Supplementary Material). A multicenter retrospective cohort study including 55 patients with post-surgical leaks and fistulae of both upper and lower GI tract treated with OverStitch reported a technical success rate of 96.7% and a clinical success rate of 63.8%. Notably, only 27% of leaks achieved long-term closure [55]. Callahan et al. reported on 24 cases of post-surgical leaks or fistulae with defects smaller than 5 cm, achieving technical success of 95.6% and clinical success of 55.6%, with higher success rates observed for esophageal and gastric defects [56]. In an Italian case series of 20 patients, endoscopic suturing of upper GI defects using OverStitch achieved 100% technical success and 80% clinical success when applied alone or in combination with SEMS or internal drainage, particularly for defects with irregular margins or associated abscess cavities [54].

#### 3.11.4. Complications

Stenosis was the most commonly reported complication, occurring in approximately 20% of cases.

### 3.12. Additional Therapies

In a substantial number of cases, endoscopic treatment is combined with adjunctive interventions to achieve clinical success. These may include video-assisted thoracoscopic surgery and pulmonary decortication, esophageal exclusion or diversion, thoracotomy, percutaneous endoscopic gastrostomy (PEG) placement, tracheal stenting, or interventional radiology drainage of abscesses [7]. Detailed analysis of these adjunctive procedures falls beyond the scope of this study.

## 4. Discussion

### 4.1. Summary of Evidence

This scoping review included 1 prospective cohort study, 30 retrospective cohort studies, 14 retrospective case series, and 1 prospective case series, encompassing a total of 2550 patients with post-surgical leaks and fistulae treated endoscopically. Additionally, 11 systematic reviews and 12 narrative reviews were included. Among the techniques evaluated (Table 3), through-the-scope (TTS) clips and tissue sealants are supported by a limited number of studies but show high efficacy in highly selected cases, such as very small leaks with well-defined margins, either used alone or in combination. TTS clips may also be useful for stent fixation, although evidence on their effectiveness in reducing stent migration is lacking. Over-the-scope clips (OTSC) represent an interesting option for defects up to 2 cm and have been applied across all gastrointestinal locations. Technical and clinical success rates vary widely and are influenced by patient- and defect-related factors, as well as operator and center experience. OTSC appears to perform better in leaks than in fistulae, particularly when applied by high-volume, experienced teams. However, adverse events and long-term clinical failures remain significant in certain settings. Endoscopic stenting is associated with high technical success and good long-term clinical outcomes, although concomitant endoscopic, surgical, or radiological interventions are often required. Stent-related adverse events are frequent, with migration being a common issue. Endoscopic vacuum therapy (EVT) is likely the most effective approach for both esophageal and colorectal leaks. The literature supporting EVT is more robust than that for other techniques, with favorable technical and clinical success rates and a reasonable complication profile. Key considerations include the need for multiple treatment sessions, longer therapy duration, and associated costs. The VAC-Stent^®^ combines the principles of stenting and EVT and appears promising; however, current evidence is limited to small case series and reports. Internal drainage (ID) has limited supporting literature but demonstrates good outcomes for post–laparoscopic sleeve gastrectomy leaks. Across different cohorts, early initiation of endoscopic therapy—particularly EVT—could emerge as one of the strongest and most consistent predictors of clinical success, underscoring the importance of prompt multidisciplinary decision-making. Endoscopic suturing is a valuable modality with high technical success, particularly in tertiary centers. Long-term efficacy, however, is limited by anatomical constraints and operator-dependent factors. Suturing is best considered in highly experienced settings, especially for defects with adequate endoluminal space and non-tangential orientation.

### 4.2. Limitations

This review has some limitations. First, the heterogeneity of study designs, the rapid evolution of endoscopic techniques, and their dependence on operator experience and individualized patient management contribute to the lack of high-quality, large, multicenter prospective studies. Moreover, as this was a scoping review, no formal assessment of methodological quality or risk of bias of the included studies was performed. Consequently, the strength of the available evidence cannot be formally graded, and the findings should be interpreted with caution. Nonetheless, the available data provide important insights and highlight the urgent need for well-designed prospective studies in this field. The exclusion of very small case series and case reports may have omitted additional publications; however, this approach improved methodological consistency and reduced heterogeneity among included studies.

### 4.3. Conclusions

Interventional endoscopy is rapidly evolving and has become an increasingly central component in the management of gastrointestinal leaks and fistulae. A broad spectrum of endoscopic techniques is now available, enabling a tailored approach based on patient characteristics, anatomical considerations, and local expertise. Despite these advances, the literature remains fragmented and is dominated by small, single-center studies and case series. Well-designed, large, multicenter studies are urgently needed to establish a more robust, evidence-based framework for the management of these common and clinically challenging conditions. 

## Figures and Tables

**Table 3:** Comparative overview of endoscopic techniques for the management of post-surgical leaks and fistulas, including indications, clinical success rates, complication profiles, and key limitations. Reported ranges/percentages reflect pooled estimates from original studies included in this review.

Technique	When to Use	Clinical Success (%)	AEs (%)	Limitations
TTS clips	Small, acute defects; regular margins; minimal cavity.	85–95 *	<5	Limited closure strength; not effective for large or chronic defects; often requires combination therapy.
OTSC	Small–moderate defects (≤20 mm); fistulas with healthy margins; post-bariatric leaks.	36–80	6–14	Difficult removal; limited efficacy if cavity present.
EVT	Large defects; anastomotic leaks with cavity; infected collections; esophageal and rectal leaks; early or intermediate phase.	71–97	0–15	Requires multiple exchanges; prolonged treatment duration; patient compliance required.
Sealants	Small chronic fistulas; adjunctive therapy; low-output tracts.	36–95	<5	Rarely effective as monotherapy.
Endoscopic suturing	Moderate-sized acute defects; accessible luminal locations; reinforcement after other therapies.	55–80	20	Technically demanding; operator-dependent; reduced efficacy in chronic settings.
SEMS	Esophageal or upper GI leaks; need for diversion; early leaks.	48–98	10–54	High complication rate; migration; need for removal; discomfort; may not control associated collections.
Internal drainage	Post-bariatric leaks; contained collections; mature cavities communicating with lumen.	75–86	<10	Limited to contained leaks; may require prolonged indwelling time; few data.
VAC-Stent^®^	Esophageal small-medium sized leaks with associated cavity. Need for simultaneous diversion.	60–77	NA (few data)	Costs; risk of late radial expansion. Few data; risk of esophageal damage or laceration.

* In specific settings, often in combination with other techniques. TTS: through-the-scope; OTSC: over-the-scope clips; EVT: endoscopic vacuum therapy; SEMS: self-expandible metal stents; GI: gastrointestinal; AEs: adverse events; NA: not available.

## Data Availability

The original contributions presented in this study are included in the article/Supplementary Material. Further inquiries can be directed to the corresponding author.

## Supplementary Materials

The following supporting information can be downloaded at: https://www.mdpi.com/article/10.3390/jcm15062291/s1, S1: Search string. Figure S1: PRISMA checklist. Table S1: Data Extraction Table—studies evaluating one signle endoscopic technique. Table S2: Data Extraction Table—studies evaluating or comparing more than one endoscopic technique.

## Abbreviations

The following abbreviations are used in this manuscript:

EVT	Endoscopic Vacuum Therapy
GI	Gastrointestinal
TTS	Through-the-scope
OTSC	Over-the-scope clip
ID	Internal drainage
SEMS	Self-expandible metal stents
