# Supplementary material for: Interventional Endoscopy for the Management of Post-Surgical Leaks and Fistulas: A Scoping Review
Source: J Clin Med. 2026 Mar 17;15(6):2291. doi: 10.3390/jcm15062291 (PMC13027318; doi:10.3390/jcm15062291)
Supplement: Supplementary file 1 [file jcm-15-02291-s001.zip › jcm-4162986-supplementary.pdf]

Supplementary Matherial

S1 - Search string:

PubMed:

("endoscopic stent"[tiab] OR "endoscopic stenting"[tiab] OR "endoscopic vacuum therapy"[tiab] OR "endoscopic negative pressure therapy"[tiab] OR "endoscopic suturing"[tiab] OR "endoscopic clipping"[tiab] OR OTSC[tiab] OR "over-the-scope clip"[tiab] OR "fibrin glue"[tiab] OR "biocompatible sealant"[tiab] OR "biocompatible scaffold"[tiab] OR "autologous fat"[tiab] OR "autologous fat tissue"[tiab]) AND ("anastomotic leak"[tiab] OR "postoperative leak"[tiab] OR "surgical leak"[tiab] OR "post-surgical leak"[tiab] OR "enteric fistula"[tiab] OR "gastrocutaneous fistula"[tiab] OR "gastrointestinal fistula"[tiab] OR "anastomotic fistula"[tiab]) AND Humans[Mesh]

Embase:

('endoscopic stent':ti,ab OR 'endoscopic stenting':ti,ab OR 'endoscopic vacuum therapy':ti,ab OR 'endoscopic negative pressure therapy':ti,ab OR 'endoscopic suturing':ti,ab OR 'endoscopic clipping':ti,ab OR 'otsc':ti,ab OR 'over-the-scope clip':ti,ab OR 'fibrin glue':ti,ab OR 'biocompatible sealant':ti,ab OR 'biocompatible scaffold':ti,ab OR 'autologous fat':ti,ab OR 'autologous fat tissue':ti,ab) AND ('anastomotic leak':ti,ab OR 'postoperative leak':ti,ab OR 'surgical leak':ti,ab OR 'post-surgical leak':ti,ab OR 'enteric

fistula':ti,ab OR 'gastrocutaneous fistula':ti,ab OR 'gastrointestinal fistula':ti,ab OR 'anastomotic fistula':ti,ab) AND [humans]/lim

Scopus:

(TITLE-ABS("endoscopic stent" OR "endoscopic stenting" OR "endoscopic vacuum therapy" OR "endoscopic negative pressure therapy" OR "endoscopic suturing" OR "endoscopic clipping" OR “OTSC” OR "over-the-scope clip" OR "fibrin glue" OR "biocompatible sealant" OR "biocompatible scaffold" OR "autologous fat" OR "autologous fat tissue"))

AND

(TITLE-ABS("anastomotic leak" OR "postoperative leak" OR "surgical leak" OR "post-surgical leak" OR "enteric fistula" OR "gastrocutaneous fistula" OR "gastrointestinal fistula" OR "anastomotic fistula"))

AND (LIMIT-TO(DOCTYPE, "ar") OR LIMIT-TO(DOCTYPE, "re"))

Figure S1 - PRISMA Checklist

Section and Topic	Item #	Checklist item	Location where item is reported
**TITLE**			
Title	1	Identify the report as a systematic review.	Lines 2-3
**ABSTRACT**			
Abstract	2	See the PRISMA 2020 for Abstracts checklist.	Lines 9-41
**INTRODUCTION**			
Rationale	3	Describe the rationale for the review in the context of existing knowledge.	Lines 48-66
Objectives	4	Provide an explicit statement of the objective(s) or question(s) the review addresses.	Lines 67-74
**METHODS**			
Eligibility criteria	5	Specify the inclusion and exclusion criteria for the review and how studies were grouped for the syntheses.	Lines 82-93
Information sources	6	Specify all databases, registers, websites, organisations, reference lists and other sources searched or consulted to identify studies. Specify the date when each source was last searched or consulted.	Lines 82-93
Search strategy	7	Present the full search strategies for all databases, registers and websites, including any filters and limits used.	Lines 94-101 + supplementary matherial
Selection process	8	Specify the methods used to decide whether a study met the inclusion criteria of the review, including how many reviewers screened each record and each report retrieved, whether they worked independently, and if applicable, details of automation tools used in the process.	Lines 102-108
Data collection process	9	Specify the methods used to collect data from reports, including how many reviewers collected data from each report, whether they worked independently, any processes for obtaining or confirming data from study investigators, and if applicable, details of automation tools used in the process.	NA for scoping reviews
Data items	10a	List and define all outcomes for which data were sought. Specify whether all results that were compatible with each outcome domain in each study were sought (e.g. for all measures, time points, analyses), and if not, the methods used to decide which results to collect.	Supplementary matherial
	10b	List and define all other variables for which data were sought (e.g. participant and intervention characteristics, funding sources). Describe any assumptions made about any missing or unclear information.	Supplementary matherial
Study risk of bias assessment	11	Specify the methods used to assess risk of bias in the included studies, including details of the tool(s) used, how many reviewers assessed each study and whether they worked independently, and if applicable, details of automation tools used in the process.	Lines 117-119: NA for scoping reviews
Effect measures	12	Specify for each outcome the effect measure(s) (e.g. risk ratio, mean difference) used in the synthesis or presentation of results.	NA for scoping reviews
Synthesis methods	13a	Describe the processes used to decide which studies were eligible for each synthesis (e.g. tabulating the study intervention characteristics and comparing against the planned groups for each synthesis (item #5)).	NA for scoping reviews
	13b	Describe any methods required to prepare the data for presentation or synthesis, such as handling of missing summary statistics, or data conversions.	NA for scoping reviews
	13c	Describe any methods used to tabulate or visually display results of individual studies and syntheses.	Line 112
	13d	Describe any methods used to synthesize results and provide a rationale for the choice(s). If meta-analysis was performed, describe the model(s), method(s) to identify the presence and extent of statistical heterogeneity, and software package(s) used.	NA for scoping reviews
	13e	Describe any methods used to explore possible causes of heterogeneity among study results (e.g. subgroup analysis, meta-regression).	NA for scoping reviews
	13f	Describe any sensitivity analyses conducted to assess robustness of the synthesized results.	NA for scoping reviews
Reporting bias assessment	14	Describe any methods used to assess risk of bias due to missing results in a synthesis (arising from reporting biases).	NA for scoping reviews

Section and Topic	Item #	Checklist item	Location where item is reported
Certainty assessment	15	Describe any methods used to assess certainty (or confidence) in the body of evidence for an outcome.	NA for scoping reviews
**RESULTS**			
Study selection	16a	Describe the results of the search and selection process, from the number of records identified in the search to the number of studies included in the review, ideally using a flow diagram.	Lines 122-124. Figure 1
	16b	Cite studies that might appear to meet the inclusion criteria, but which were excluded, and explain why they were excluded.	NA
Study characteristics	17	Cite each included study and present its characteristics.	Paragraph 3
Risk of bias in studies	18	Present assessments of risk of bias for each included study.	NA for scoping reviews
Results of individual studies	19	For all outcomes, present, for each study: (a) summary statistics for each group (where appropriate) and (b) an effect estimate and its precision (e.g. confidence/credible interval), ideally using structured tables or plots.	NA for scoping reviews
Results of syntheses	20a	For each synthesis, briefly summarise the characteristics and risk of bias among contributing studies.	NA for scoping reviews
	20b	Present results of all statistical syntheses conducted. If meta-analysis was done, present for each the summary estimate and its precision (e.g. confidence/credible interval) and measures of statistical heterogeneity. If comparing groups, describe the direction of the effect.	NA for scoping reviews
	20c	Present results of all investigations of possible causes of heterogeneity among study results.	NA for scoping reviews
	20d	Present results of all sensitivity analyses conducted to assess the robustness of the synthesized results.	NA for scoping reviews
Reporting biases	21	Present assessments of risk of bias due to missing results (arising from reporting biases) for each synthesis assessed.	NA for scoping reviews
Certainty of evidence	22	Present assessments of certainty (or confidence) in the body of evidence for each outcome assessed.	NA for scoping reviews
**DISCUSSION**			
Discussion	23a	Provide a general interpretation of the results in the context of other evidence.	Section: discussion
	23b	Discuss any limitations of the evidence included in the review.	Section: discussion
	23c	Discuss any limitations of the review processes used.	Section: discussion
	23d	Discuss implications of the results for practice, policy, and future research.	Section: discussion
**OTHER INFORMATION**			
Registration and protocol	24a	Provide registration information for the review, including register name and registration number, or state that the review was not registered.	Lines 80-81
	24b	Indicate where the review protocol can be accessed, or state that a protocol was not prepared.	Lines 80-81
	24c	Describe and explain any amendments to information provided at registration or in the protocol.	NA
Support	25	Describe sources of financial or non-financial support for the review, and the role of the funders or sponsors in the review.	Line 559
Competing interests	26	Declare any competing interests of review authors.	Line 567
Availability of data, code and other materials	27	Report which of the following are publicly available and where they can be found: template data collection forms; data extracted from included studies; data used for all analyses; analytic code; any other materials used in the review.	Line 562-563

From: Page MJ, McKenzie JE, Bossuyt PM, Boutron I, Hoffmann TC, Mulrow CD, et al. The PRISMA 2020 statement: an updated guideline for reporting systematic reviews. BMJ 2021;372:n71. doi: 10.1136/bmj.n71. This work is licensed under CC BY 4.0. To view a copy of this license, visit https://creativecommons.org/licenses/by/4.0/

Table S1 and S2: Legenda

PC: prospective cohort; RC: retrospective cohort; CS: case series; PCS: prospective case series; SR: systematic review; NR: narrative review; ET: esophagotracheal; EB: esophagobronchial; EP: esophagopulmonary, VATS: video assisted thoracoscopy; EVT: endoscopic vacuum therapy; SEMS: self-expandible metal stents; TTS: through-the-scope; OTSC: over-the-scope clips; ID: internal drainage; IR: interventional radiology: PEG: percutaneous endoscopic gastrostomy.

Table S1 - Data Extraction Table – studies evaluating one single endoscopic technique

Author and year	Study Design	N	Leak / Fistula	Site	Technique	Number of Sessions	Additional Therapies	Technical Success	Clinical Success	Time to Closure (days)	Need for Reintervention	Complications	Death
Maier 2023 [1]	RC	37	Leaks	Esophagus	EVT	5	35% (stent)	NA	71%	NA	29%	NA	6%
Chon 2023 [2]	CS	20	Leaks	Esophagus	EVT	2	Stent (20%)	100%	75% EVT, 95% overall	12	25%	0%	0%
Lee 2023 [3]	RC	21	Leaks	Esophagus	EVT	3	NA	NA	91%	16	5%	14.3% strictures	0.00%
Momblan 2023 [4]	PC	102	Leaks + perforation	Esophagus, stomach	EVT	5	NA	NA	82%	19	NA	10.7% strictures, 5.9% other	19.60 %
Reimer 2022 [5]	RC	156	Leak	Esophagus, stomach	EVT	5	23.40%	NA	91%	14	29.90%	12%	3.90%
Kühn 2022 [6]	RC	57	Leak	colorectal	EVT	7	0%	100%	93%	21	0%	NA	4%
Hayami 2021 [7]	CS	23	Leak	Esophagus	EVT	3	SEMS (13%)	100%	73.90%	17	NA	8.7% EB / EP fistula	13%
Jung 2021 [8]	CS	23	Leak	Esophagus	EVT	3.4	SEMS (8.7%), fibrin glue (4.3%)	100%	78%	15.7	0%	7% bleeding	NA
Kühn 2020 [9]	RC	21	leak	Colorectal	EVT	NA	NA	NA	85.70%	42	NA	NA	3.60%
Min 2019 [10]	RC	20	Leak	Esophagus	EVT	5	NA	NA	95%	14.5	NA	NA	5%
Mencio 2018 [11]	CS	49	Leak, perforations	Esophagus, stomach, small bowel, colorectal	EVT	2.7 (small bowel) 10.5 (gastric)	NA	100%	85.7% (100% esophageal, 60% colorectal)	13.7-55.3	14%	0%	2%
Bludau 2018 [12]	CS	77	Leak, perforation	Esophagus	EVT	2.75	SEMS (27%)	NA	77.90%	11	NA	Hemorrage, MOF, ARDS	15.60 %
Laukoetter 2017 [13]	CS	39	Leak	Esophagus, stomach	EVT	6	OTSC (17.9%)	NA	94.2% (overall, including	NA	NA	10.2% stenosis	12.90 %

									perforation s)				
Kuehn 2016 [14]	CS	20	Leak	Colorectal	EVT	6	NA	100%	90%	NA	NA	15% stenosis, 5% bleeding.	NA
Haruštiak 2020 [15]	CS	39	Leak	Esophagus	SEMS	NA	23% IR, 7.6% EVT	NA	64%	NA	21% reoperated	10% airway fistula	10%
Azevedo 2020 [15]	RC	53	Leak	Esophagus, stomach	SEMS	NA	3.7% (NA)	100%	62.30%	81.9	13%	NA	20.30 %
El Hajj 2014 [17]	RC	54	Leak, fistula	Esophagus	SEMS	NA	27.8% (VATS and pulmonary decortication, esophageal exclusion / diversion, thoracotomy, PEG, tracheal stent, IR)	100%	83%	46.7	9.30%	54% dysphagia / chest pain / bleeding / pneumothorax / migration / tissue ingrowth	1.80%
Freeman 2012 [18]	RC	187	Leak, fistula, perforati on	Esophagus	SEMS	NA	NA	NA	92%	NA	8%	Stent migration 17%	2.10%
van Boeckel 2012 [19]	CS	52	Leak, perforati on	Esophagus	SEMS, SEPS	NA	NA	99% positioning , 89% removal	76% (PSEMS 73%, FSEMS 83%, SEPS 83%)	39	7.60%	46% (ingrowth, obstruction, migration, hemorrage, esophageal rupture during removal, severe retrosternal pain)	13.40 %
Dai 2009 [20]	CS	22	Leak	Esophagus	SEPS	1 (40.9%), >3 (13.6%)	Interventiona l drainage (45%)	100%	95.40%	23	9%	22.7% migration	4.50%
Zhang 2022 [21]	RC	98	Fistula (definiti on lacking)	Miscellaneo us	OTSC	NA	NA	100%	55.10%	NA	NA	NA	NA
Morrell 2020 [22]	RC	117	Leak, fistula	Miscellaneo us	OTSC		19.7% other endoscopy	NA	66.10%	NA	11.10%	NA	3.60%
Donatelli 2016 [23]	CS	30	Leak, fistula	Miscellaneo us	OTSC	1	NA	50%	36.60%	NA	23.30%	6.6% migration / stricture	NA
Mercky 2015 [24]	RC	34	Leak, fistula	Miscellaneo us	OTSC	1	26.7% other endoscopy (cyanoacrylat e, SEMS or standard clips)	88.20%	53.00%	NA	26.70%	14.7% intraprocedural	

Haito-Chavez 2014 [25]	RC	161	Leak, fistula, perforation	Miscellaneous	OTSC	NA	APC	93.80%	60.20%	NA	NA	NA	NA
Chon 2022 [26]	PCS	20	Leak, perforation	Esophagus	VACstent	1.2	4.8	100	60	4.8	NA	0%	0%
Granata 2020 [27]	RC	20	Leak, fistula	Esophagus, stomach	Endoscopic suturing	NA	SEMS (55%)	100%	80%	NA	NA	20% stenosis	NA
Callahan 2019 [28]	RC	24	Leak, fistula	Miscellaneous	Endoscopic suturing	1	NA	95.80%	55.60%	NA	NA	NA	NA
Sharaiha 2016 [29]	RC	55	Leak, fistula	Miscellaneous	Endoscopic suturing	NA	NA	96.70%	63.80%	NA	NA	NA	NA
Donatelli 2015 [30]	CS	67	Leaks	Stomach	ID	3.14	NA	99%	74.60%	57.5	NA	8.9% stenosis	3%
Lippert 2011 [31]	CS	52	Leak, fistula	Miscellaneous	Sealants	4	53.9% (clipping, hystoacryl, stent, stitching, rinsing, draining)	NA	36.50%	70	34.60%	46.3% abscess, 30.8% mediastinitis / peritonitis, 28.8% sepsis	21.10 %

Table S2: Data Extraction Table – studies evaluating or comparing more than one endoscopic technique

Author and year	Study Design	N	Leak / Fistula	Site	Technique	Number of Sessions	Additional Therapies	Technical Success	Clinical Success	Time to Closure (days)	Need for Reintervention	Complications	Death
Triantafyllou 2025 [31]	RC	29	Leak	Esophagus	SEMS, EVT	NA	NA	NA	97%	NA	36% after stent	NA	3%
Ascari 2024 [33]	RC	47	Leak	Esophagus	EVT, SEMS, TTS	NA	NA	NA	45.2% (first line), 87.1% (all line)	NA	NA	NA	11.30 %
Rosianu 2024 [34]	RC	55	Fistula	Esophagus	SEMS, TTS	NA	NA	NA	98%	NA	NA	20% (migration, perforation, hemorrage, mediastinitis)	5%
Eichelmann 2021 [35]	RC	38	Leak	Esophagus	EVT vs. SEMS	7.4 vs. 1.5	NA	NA	NA	30 vs. 44	NA	NA	NA
Zhong 2021 [36]	RC	22	Leak	Esophagus	TTS, sealants	NA	NA	100.00%	95.50%	37	NA	4.55%	4.55%

Kantowski 2020 [37]	RC	89	leak	Colorectal	EVT +/- transanal rinsing therapy	6	NA	NA	58-84%	70	3-35%	12% bleeding / fistula / stenosis	2-7%
Berlth 2019 [38]	RC	111	Leak	Esophagus, stomach	SEMS vs. EVT	1 vs. 3	NA	74% vs. 85%	85.7% vs 72.4%	27 vs. 12	NA	SEMS: 20% dislocation, 4.3% leak persistence, 6.5% ET fistula, 7.2% stenosis, 1.4% perforation. EVT: 15% dislocation, 2.9% stenosis.	NA
Baltin 2019 [39]	RC	39	Leak	Esophagus	Stent vs. EVT	NA	NA	NA	82%	31	NA	NA	NA
Lorenzo 2018 [40]	RC	100	Fistula	Stomach (sleeve)	SEMS, OTSC, NCD, sealants vs ID	3.7	NA	NA	86% overall, 45% OTSC, 86% ID, 63% clip/SEMS	NA	6%	50% stent migration / marginal ulcers	2%
Xu 2016 [41]	RC	28	Leak	Esophagus	TTS + NCD	NA	NA	NA	85.70%	NA	NA	NA	14.30 %
Mennigen 2015 [42]	RC	45	Leak	Esophagus	SEMS vs. EVT	1 for FSEMS vs. 6.5 for EVT	23.3% switch from FSEMS to EVT	100%	63.3% vs. 93.3%	36 vs 26.5	13.3% FSEMS	NA	26.7% vs 6.7%
Manta 2015 [43]	CS	76	Leak	Miscellaneous	SEMS, OTSC, sealants, EVT	NA	NA	NA	80.30%	NA	19.70%	NA	1.30%
Schniewind 2013 [44]	RC	62	leak	Esophagus	Surgery vs SEMS/SEPS vs EVT vs conservative	NA	NA	NA	NA	NA	NA	NA	50% (surgery) vs 12 % (EVT) vs 83% (STENT)
Brangewitz 2013 [45]	RC	71	Leak	Esophagus	SEMS vs EVT	3 vs. 7	NA	100%	53.8% vs. 84.4%	33 vs. 23	NA	stricture (28.2% vs. 9.4%), bleeding (7.7% vs. 3.1%), EB fistula (0% vs 3.1%), esophageal necrosis (3.2% vs. 0%)	25.2% vs. 15.6%
Lee 2013 [3]	RC	55	Leak	Stomach	TTS, sealants	NA	NA	95%	95%	NA	5%	0%	0%
Schaheen 2014 [46]	SR												

van Halsema 2015 [47]	SR												
Arezzo 2017 [48]	SR												
Clifford 2019 [49]	SR												
Bartell 2020 [50]	SR												
de Lacy 2022 [51]	SR												
Wang 2022 [52]	SR												
Scognamiglio 2022 [53]	SR												
Bawa 2023 [54]	SR												
Vohra 2025 [55]	SR												
Kehagias 2025 [56]	SR												
Chan 2022 [57]	NR												
Dell’Anna 2024 [58]	NR												
Ardila 2025 [59]	NR												
Famiglietti 2020 [60]	NR												
Bhurwal 2020 [61]	NR												
Kumar 2014 [62]	NR												
Willingham 2015 [63]	NR												
Kobara 2019 [64]	NR												
Pines 2018 [65]	NR												
Guida 2022 [66]	NR												
Kouladouros 2024 [67]	NR												
Cereatti 2020 [68]	NR												

1. Maier, J., Kandulski, A., Donlon, N. E., Werner, J. M., Mehrl, A., Müller, M., Doenecke, A., Schlitt, H. J., Hornung, M., Weiss, A. R. R. Endoscopic vacuum therapy significantly improves clinical outcomes of anastomotic leakages after 2-stage, 3-stage, and transhiatal esophagectomies. *Langenbecks Arch. Surg.* **2023**, *408*, 90. https://doi.org/10.1007/s00423-023-02826-3.

2. Chon, S. H., Brunner, S., Müller, D. T., Lorenz, F., Stier, R., Streller, L., Eckhoff, J., Straatman, J., Babic, B., Schiffmann, L. M., Schröder, W. Time to endoscopic vacuum therapy-lessons learned after > 150 robotic-assisted minimally invasive esophagectomies (RAMIE) at a German high-volume center. *Surg. Endosc.* **2023**, *37*, 741–748. https://doi.org/10.1007/s00464-022-09754-1.
3. Lee, S.; Ahn, J.Y.; Jung, H.Y.; Choi, K. S.; Kim, D. H.; Choi, K. D.; Song, H. J.; Lee, G. H.; Kim, J. H.; Kim, B. S. et al. Clinical outcomes of endoscopic and surgical management for postoperative upper gastrointestinal leakage. *Surg. Endosc.* **2013**, *27*, 4232–4240. https://doi.org/10.1007/s00464-013-3028-y.
4. Momblan, D., Gimeno Garcia, A. Z., Busquets, D., Juzgado, D., García Lledó, J., Ferrero, E., Tejedor-Tejada, J., Junquera, F., Díaz-Tasende, J., Moris, M. et al. Endoscopic Vacuum Therapy for Upper Gastrointestinal Leaks and Perforations: Analysis From a Multicenter Spanish Registry. *Am. J. Gastroenterol.* **2023**, *118*, 1797–1806. https://doi.org/10.14309/ajg.0000000000002475.
5. Reimer, S., Seyfried, F., Flemming, S., Brand, M., Weich, A., Widder, A., Plaßmeier, L., Kraus, P., Döring, A., Hering, I. et al. Evolution of endoscopic vacuum therapy for upper gastrointestinal leakage over a 10-year period: A quality improvement study. *Surg. Endosc.* **2022**, *36*, 9169–9178. https://doi.org/10.1007/s00464-022-09400-w.
6. Kühn, F., Hasenhütl, S. M., Hofmann, F. O., Wirth, U., Drefs, M., Werner, J., Schiergens, T. S. Endoscopic Vacuum Therapy for Left-Sided Colorectal Anastomotic Leak Without Fecal Diversion. *Dis. Colon. Rectum.* **2022**, *65*, 421–428. https://doi.org/10.1097/DCR.0000000000001959.
7. Hayami, M., Klevebro, F., Tsekrekos, A., Samola Winnberg, J., Kamiya, S., Rouvelas, I., Nilsson, M., Lindblad, M. Endoscopic vacuum therapy for anastomotic leak after esophagectomy: a single-center's early experience. *Dis Esophagus*. 2021;34(9):doaa122. doi:10.1093/dote/doaa122
8. Jung, C. F. M., Müller-Dornieden, A., Gaedcke, J., Kunsch, S., Gromski, M. A., Biggemann, L., Seif Amir Hosseini, A., Ghadimi, M., Ellenrieder, V., Wedi, E. Impact of Endoscopic Vacuum Therapy with Low Negative Pressure for Esophageal Perforations and Postoperative Anastomotic Esophageal Leaks. *Digestion*. 2021;102(3):469-479. doi:10.1159/000506101
9. Kühn, F., Janisch, F., Schwandner, F., Gock, M., Wedermann, N., Witte, M., Klar, E., Schiffmann, L.. Comparison Between Endoscopic Vacuum Therapy and Conventional Treatment for Leakage After Rectal Resection. *World J. Surg.* **2020**, *44*, 1277–1282. https://doi.org/10.1007/s00268-019-05349-5.
10. Min, Y. W., Kim, T., Lee, H., Min, B. H., Kim, H. K., Choi, Y. S., Lee, J. H., Rhee, P. L., Kim, J. J., Zo, J. I. et al. Endoscopic vacuum therapy for postoperative esophageal leak. *BMC Surg.* **2019**, *19*, 37. https://doi.org/10.1186/s12893-019-0497-5.
11. Mencio MA, Ontiveros E, Burdick JS, Leeds SG. Use of a novel technique to manage gastrointestinal leaks with endoluminal negative pressure: a single institution experience. *Surg Endosc*. 2018;32(7):3349-3356. doi:10.1007/s00464-018-6055-x
12. Bludau, M., Fuchs, H. F., Herbold, T., Maus, M. K. H., Alakus, H., Popp, F., Leers, J. M., Bruns, C. J., Hölscher, A. H., Schröder, W. et al. Results of endoscopic vacuum-assisted closure device for treatment of upper GI leaks. *Surg. Endosc.* **2018**, *32*, 1906–1914. https://doi.org/10.1007/s00464-017-5883-4.
13. Laukoetter, M. G., Mennigen, R., Neumann, P. A., Dhayat, S., Horst, G., Palmes, D., Senninger, N., Vowinkel, T. Successful closure of defects in the upper gastrointestinal tract by endoscopic vacuum therapy (EVT): A prospective cohort study. *Surg. Endosc.* **2017**, *31*, 2687–2696. https://doi.org/10.1007/s00464-016-5265-3.
14. Kuehn, F., Janisch, F., Schwandner, F., Alsfasser, G., Schiffmann, L., Gock, M., Klar, E. Endoscopic Vacuum Therapy in Colorectal Surgery. *J Gastrointest Surg*. 2016;20(2):328-334. doi:10.1007/s11605-015-3017-7
15. Haruštiak, T., Tvrdoň, J., Pazdro, A., Šnajdauf, M., Faltová, H., Teršíp, T., Wolesky, J., Černý, V., Schützner, J., Stolz, A. et al. Treatment of anastomotic leak after Ivor Lewis esophagectomy. Léčba insuficience hrudní anastomózy po Ivor Lewis ezofagektomii. *Rozhl Chir*. 2020;99(10):438-446.
16. Azevedo, R., Coelho, M., Gouveia, C., Loureiro, R., Freire, R., Silva, R., Cravo, M., Pereira, A. D., Ribeiro, M. D., de Ferro, S. M. Clinical score predicting a successful endoscopic approach of esophageal anastomotic leaks: External validation. *Eur. J. Gastroenterol. Hepatol.* **2020**, *32*, 490–495. https://doi.org/10.1097/MEG.0000000000001621.
17. El Hajj, I. I., Imperiale, T. F., Rex, D. K., Ballard, D., Kesler, K. A., Birdas, T. J., Fatima, H., Kessler, W. R., DeWitt, J. M. Treatment of esophageal leaks, fistulae, and perforations with temporary stents: Evaluation of efficacy, adverse events, and factors associated with successful outcomes. *Gastrointest. Endosc.* **2014**, *79*, 589–598. https://doi.org/10.1016/j.gie.2013.08.039.
18. Freeman, R.K., Ascioti, A.J., Giannini, T., Mahidhara, R.J. Analysis of unsuccessful esophageal stent placements for esophageal perforation, fistula, or anastomotic leak. *Ann. Thorac. Surg.* **2012**, *94*, 959–965. https://doi.org/10.1016/j.athoracsur.2012.05.047.

19. van Boeckel, P. G., Dua, K. S., Weusten, B. L., Schmits, R. J., Surapaneni, N., Timmer, R., Vleggaar, F. P., Siersema, P. D. Fully covered self-expandable metal stents (SEMS), partially covered SEMS and self-expandable plastic stents for the treatment of benign esophageal ruptures and anastomotic leaks. *BMC Gastroenterol.* **2012**, *12*, 19. https://doi.org/10.1186/1471-230X-12-19.
20. Dai, Y.Y., Gretschel, S., Dudeck, O., Rau, B., Schlag, P.M., Hünerbein, M. Treatment of oesophageal anastomotic leaks by temporary stenting with self-expanding plastic stents. *Br. J. Surg.* **2009**, *96*, 887–891. https://doi.org/10.1002/bjs.6648.
21. Zhang, J., Da, B., Diao, Y., Qian, X., Wang, G., Gu, G., Wang, Z. Efficacy and safety of over-the-scope clips (OTSC®) for closure of gastrointestinal fistulas less than 2 cm. *Surg. Endosc.* **2022**, *36*, 5267–5274. https://doi.org/10.1007/s00464-021-08904-1.
22. Morrell, D. J., Winder, J. S., Johri, A., Docimo, S., Juza, R. M., Witte, S. R., Alli, V. V., & Pauli, E. M. Over-the-scope clip management of non-acute, full-thickness gastrointestinal defects. *Surg. Endosc.* **2020**, *34*, 2690–2702. https://doi.org/10.1007/s00464-019-07030-3.
23. Donatelli, G., Cereatti, F., Dhumane, P., Vergeau, B. M., Tuszynski, T., Marie, C., Dumont, J. L., Meduri, B. Closure of gastrointestinal defects with Ovesco clip: Long-term results and clinical implications. *Ther. Adv. Gastroenterol.* **2016**, *9*, 713–721. https://doi.org/10.1177/1756283X16652325.
24. Mercky, P., Gonzalez, J. M., Aimore Bonin, E., Emungania, O., Brunet, J., Grimaud, J. C., Barthet, M. Usefulness of over-the-scope clipping system for closing digestive fistulas. *Dig. Endosc.* **2015**, *27*, 18–24. https://doi.org/10.1111/den.12295.
25. Haito-Chavez, Y., Law, J. K., Kratt, T., Arezzo, A., Verra, M., Morino, M., Sharaiha, R. Z., Poley, J. W., Kahaleh, M., Thompson, C. C. et al. International multicenter experience with an over-the-scope clipping device for endoscopic management of GI defects (with video). *Gastrointest. Endosc.* **2014**, *80*, 610–622. https://doi.org/10.1016/j.gie.2014.03.049.
26. Chon, S. H., Scherdel, J., Rieck, I., Lorenz, F., Dratsch, T., Kleinert, R., Gebauer, F., Fuchs, H. F., Goeser, T., Bruns, C. J. A new hybrid stent using endoscopic vacuum therapy in treating esophageal leaks: A prospective single-center experience of its safety and feasibility with mid-term follow-up. *Dis. Esophagus* **2022**, 35, doab067. https://doi.org/10.1093/dote/doab067.
27. Granata, A., Amata, M., Ligresti, D., Martino, A., Tarantino, I., Barresi, L., & Traina, M. Endoscopic management of post-surgical GI wall defects with the overstitch endosuturing system: A single-center experience. *Surg. Endosc.* **2020**, *34*, 3805–3817. https://doi.org/10.1007/s00464-019-07145-7.
28. Callahan, Z. M., Su, B., Kuchta, K., Conaty, E., Novak, S., Linn, J., Murad, F. M., Carbray, J., Ujiki, M. Endoscopic Suturing Results in High Technical and Clinical Success Rates for a Variety of Gastrointestinal Pathologies. *J. Gastrointest. Surg.* **2020**, *24*, 278–287. https://doi.org/10.1007/s11605-019-04485-6.
29. Sharaiha, R. Z., Kumta, N. A., DeFilippis, E. M., Dimaio, C. J., Gonzalez, S., Gonda, T., Rogart, J., Siddiqui, A., Berg, P. S., Samuels, P. et al. A Large Multicenter Experience With Endoscopic Suturing for Management of Gastrointestinal Defects and Stent Anchorage in 122 Patients: A Retrospective Review. *J. Clin. Gastroenterol.* **2016**, *50*, 388–392. https://doi.org/10.1097/MCG.0000000000000336.
30. Donatelli, G., Dumont, J. L., Cereatti, F., Ferretti, S., Vergeau, B. M., Tuszynski, T., Pourcher, G., Tranchart, H., Mariani, P., Meduri, A. Treatment of Leaks Following Sleeve Gastrectomy by Endoscopic Internal Drainage (EID). *Obes. Surg.* **2015**, *25*, 1293–1301. https://doi.org/10.1007/s11695-015-1675-x.
31. Lippert, E., Klebl, F. H., Schweller, F., Ott, C., Gelbmann, C. M., Schölmerich, J., Endlicher, E., Kullmann, F. Fibrin glue in the endoscopic treatment of fistulae and anastomotic leakages of the gastrointestinal tract. *Int. J. Color. Dis.* **2011**, *26*, 303–311. https://doi.org/10.1007/s00384-010-1104-5.
32. Triantafyllou, A., Mela, E., Theodoropoulos, C., Theodorou, A. P., Kitsou, E., Saliaris, K., Katsila, S., Kakounis, K., Triantafyllou, T., Theodorou, D. Addressing Anastomotic Leak After Esophagectomy: Insights from a Specialized Unit. *J Clin Med*. 2025;14(11):3694. Published 2025 May 25. doi:10.3390/jcm14113694
33. Ascari, F.; De Pascale, S.; Rosati, R.; Giacopuzzi, S.; Puccetti, F.; Weindelmayer, J.; Cusin, S.; Leone, B.; Fumagalli Romario, U. Multicenter study on the incidence and treatment of mediastinal leaks after esophagectomy (MuMeLe 2). *J. Gastrointest. Surg.* **2024**, *28*, 1072–1077. https://doi.org/10.1016/j.gassur.2024.04.024.
34. Rosianu, C.G.; Pușcașu, A.; Hoara, P.; Predescu, D., Birla, R., Achim, F., Strimbu, V. C., Constantinoiu, S., Andronic, O., Constantinescu, A. Efficiency and Complications of Esophageal Stenting in the Management of Postoperative Fistulas. *J. Clin. Med.* **2024**, *13*, 6167. https://doi.org/10.3390/jcm13206167.
35. Eichelmann, A. K., Ismail, S., Merten, J., Slepecka, P., Palmes, D., Laukötter, M. G., Pascher, A., Mardin, W. A.Economic Burden of Endoscopic Vacuum Therapy Compared to Alternative Therapy Methods in Patients with Anastomotic Leakage After Esophagectomy. *J. Gastrointest. Surg.* **2021**, *25*, 2447–2454. https://doi.org/10.1007/s11605-021-04955-w.

36. Zhong, L., Zhong, J., Tan, Z., Wei, Y., Su, X., Wen, Z., Rong, T., Hu, Y., Luo, K. An Approach to Accelerate Healing and Shorten the Hospital Stay of Patients With Anastomotic Leakage After Esophagectomy: An Explorative Study of Systematic Endoscopic Intervention. *Front. Oncol.* **2021**, *11*, 657955. https://doi.org/10.3389/fonc.2021.657955.
37. Kantowski, M., Kunze, A., Bellon, E., Rösch, T., Settmacher, U., Tachezy, M. Improved colorectal anastomotic leakage healing by transanal rinsing treatment after endoscopic vacuum therapy using a novel patient-applied rinsing catheter. *Int. J. Color. Dis.* **2020**, *35*, 109–117. https://doi.org/10.1007/s00384-019-03456-2.
38. Berlth, F., Bludau, M., Plum, P. S., Herbold, T., Christ, H., Alakus, H., Kleinert, R., Bruns, C. J., Hölscher, A. H., Chon, S. H.Self-Expanding Metal Stents Versus Endoscopic Vacuum Therapy in Anastomotic Leak Treatment After Oncologic Gastroesophageal Surgery. *J. Gastrointest. Surg.* **2019**, *23*, 67–75. https://doi.org/10.1007/s11605-018-4000-x.
39. Baltin, C., Kron, F., Urbanski, A., Zander, T., Kron, A., Berlth, F., Kleinert, R., Hallek, M., Hoelscher, A. H., Chon, S. H. The economic burden of endoscopic treatment for anastomotic leaks following oncological Ivor Lewis esophagectomy. *PLoS ONE* **2019**, *14*, e0221406. https://doi.org/10.1371/journal.pone.0221406.
40. Lorenzo, D., Guilbaud, T., Gonzalez, J. M., Benezech, A., Dutour, A., Boullu, S., Berdah, S., Bège, T., Barthet, M. Endoscopic treatment of fistulas after sleeve gastrectomy: A comparison of internal drainage versus closure. *Gastrointest. Endosc.* **2018**, *87*, 429–437. https://doi.org/10.1016/j.gie.2017.07.032.
41. Xu, K.; Chen, S.; Bian, W.; Xie, H.; Ma, H.; Ni, B. Digital subtraction angiography-guided esophagography, intraluminal drainage, and endoscopic clipping-complex managements for intrathoracic esophagogastric anastomotic leak. *J. Surg. Res.* **2016**, *204*, 68–74. https://doi.org/10.1016/j.jss.2016.04.018.
42. Mennigen, R., Harting, C., Lindner, K., Vowinkel, T., Rijcken, E., Palmes, D., Senninger, N., Laukoetter, M. G. Comparison of Endoscopic Vacuum Therapy Versus Stent for Anastomotic Leak After Esophagectomy. *J Gastrointest Surg*. 2015;19(7):1229-1235. doi:10.1007/s11605-015-2847-7
43. Manta, R., Caruso, A., Cellini, C., Sica, M., Zullo, A., Mirante, V. G., Bertani, H., Frazzoni, M., Mutignani, M., Galloro, G. et al. Endoscopic management of patients with post-surgical leaks involving the gastrointestinal tract: A large case series. *United Eur. Gastroenterol. J.* **2016**, *4*, 770–777. https://doi.org/10.1177/2050640615626051.
44. Schniewind, B., Schafmayer, C., Voehrs, G., Egberts, J., von Schoenfels, W., Rose, T., Kurdow, R., Arlt, A., Ellrichmann, M., Jürgensen, C.et al. Endoscopic endoluminal vacuum therapy is superior to other regimens in managing anastomotic leakage after esophagectomy: A comparative retrospective study. *Surg. Endosc.* **2013**, *27*, 3883–3890. https://doi.org/10.1007/s00464-013-2998-0.
45. Brangewitz, M., Voigtländer, T., Helfritz, F. A., Lankisch, T. O., Winkler, M., Klempnauer, J., Manns, M. P., Schneider, A. S., Wedemeyer, J. Endoscopic closure of esophageal intrathoracic leaks: Stent versus endoscopic vacuum-assisted closure, a retrospective analysis. *Endoscopy* **2013**, *45*, 433–438. https://doi.org/10.1055/s-0032-1326435.
46. Schaheen, L., Blackmon, S.H., Nason, K.S. Optimal approach to the management of intrathoracic esophageal leak following esophagectomy: A systematic review. *Am. J. Surg.* **2014**, *208*, 536–543. https://doi.org/10.1016/j.amjsurg.2014.05.011.
47. van Halsema EE, van Hooft JE. Clinical outcomes of self-expandable stent placement for benign esophageal diseases: A pooled analysis of the literature. *World J Gastrointest Endosc*. 2015;7(2):135-153. doi:10.4253/wjge.v7.i2.135
48. Arezzo, A., Bini, R., Lo Secco, G., Verra, M., Passera, R. The role of stents in the management of colorectal complications: A systematic review. *Surg. Endosc.* **2017**, *31*, 2720–2730. https://doi.org/10.1007/s00464-016-5315-x.
49. Clifford RE, Fowler H, Govindarajah N, Vimalachandran D, Sutton PA. Early anastomotic complications in colorectal surgery: a systematic review of techniques for endoscopic salvage. *Surg Endosc*. 2019;33(4):1049-1065. doi:10.1007/s00464-019-06670-9
50. Bartell, N., Bittner, K., Kaul, V., Kothari, T.H., Kothari, S. Clinical efficacy of the over-the-scope clip device: A systematic review. *World J. Gastroenterol.* **2020**, *26*, 3495–3516. https://doi.org/10.3748/wjg.v26.i24.3495.
51. de Lacy, F. B., Talboom, K., Roodbeen, S. X., Blok, R., Curell, A., Tanis, P. J., Bemelman, W. A., Hompes, R. Endoscopic vacuum therapy and early surgical closure after pelvic anastomotic leak: Meta-analysis of bowel continuity rates. *Br. J. Surg.* **2022**, *109*, 822–831. https://doi.org/10.1093/bjs/znac158.
52. Wang, Q., Li, J., Liu, S., Fang, C., Chen, W. Efficacy and safety of over-the-scope clips for colorectal leaks and fistulas: A pooled analysis. *Minim. Invasive Ther. Allied Technol.* **2022**, *31*, 825–834. https://doi.org/10.1080/13645706.2021.2010218.
53. Scognamiglio, P., Reeh, M., Melling, N., Kantowski, M., Eichelmann, A. K., Chon, S. H., El-Sourani, N., Schön, G., Höller, A., Izbicki, J. R., & Tachezy, M. Management of intra-thoracic anastomotic leakages after esophagectomy: Updated systematic review and meta-analysis of endoscopic vacuum therapy versus stenting. *BMC Surg.* **2022**, *22*, 309. https://doi.org/10.1186/s12893-022-01764-z.

54. Bawa JH, Sulutaura L, Patel NM, Sufi PA, Parmar C. Closure of Gastrocutaneous Fistula Following Bariatric Surgery: a Systematic Review. *Obes Surg*. 2023;33(11):3658-3668. doi:10.1007/s11695-023-06861-3
55. Vohra, I., Gopakumar, H., Sharma, N.R., Puli, S.R. Efficacy of endoscopic vacuum therapy in esophageal luminal defects: A systematic review and meta-analysis. *Clin. Endosc.* **2025**, *58*, 53–62. https://doi.org/10.5946/ce.2023.282.
56. Kehagias, D., Abogabal, S., Lampropoulos, C., Haider, M. I., Kehagias, I., Jain, P., Wong, V. VacStent as a novel therapeutic approach for esophageal perforations and anastomotic leaks- a systematic review of the literature. *BMC Surg.* **2025**, *25*, 309. https://doi.org/10.1186/s12893-025-03067-5.
57. Chan SM, Auyeung KKY, Lam SF, Chiu PWY, Teoh AYB. Current status in endoscopic management of upper gastrointestinal perforations, leaks and fistulas. *Dig Endosc*. 2022;34(1):43-62. doi:10.1111/den.14061
58. Dell'Anna G., Fanti L., Fanizza J., Barà, R., Barchi, A., Fasulo, E., Elmore, U., Rosati, R., Annese, V., Laterza, L. et al. VAC-Stent in the Treatment of Post-Esophagectomy Anastomotic Leaks: A New "Kid on the Block" Who Marries the Best of Old Techniques-A Review. *J Clin Med*. 2024;13(13):3805. Published 2024 Jun 28. doi:10.3390/jcm13133805
59. Ardila CM, González-Arroyave D, Ramírez-Arbeláez J. Comprehensive Umbrella Review of the Management of Esophageal Anastomotic Leaks. *J Clin Med*. 2025;14(9):2881. Published 2025 Apr 22. doi:10.3390/jcm14092881
60. Famiglietti A, Lazar JF, Henderson H, Hamm, M., Malouf, S., Margolis, M., Watson, T. J., & Khaitan, P. G. Management of anastomotic leaks after esophagectomy and gastric pull-up. *J Thorac Dis*. 2020;12(3):1022-1030. doi:10.21037/jtd.2020.01.15
61. Bhurwal A, Mutneja H, Tawadross A, Pioppo L, Brahmbhatt B. Gastrointestinal fistula endoscopic closure techniques. *Ann Gastroenterol*. 2020;33(6):554-562. doi:10.20524/aog.2020.0543
62. Kumar N, Larsen MC, Thompson CC. Endoscopic Management of Gastrointestinal Fistulae. *Gastroenterol Hepatol (N Y)*. 2014;10(8):495-452.
63. Willingham, F.F., Buscaglia, J.M. Endoscopic Management of Gastrointestinal Leaks and Fistulae. *Clin. Gastroenterol. Hepatol.* **2015**, *13*, 1714–1721. https://doi.org/10.1016/j.cgh.2015.02.010.
64. Kobara H, Mori H, Nishiyama N, Fujihara, S., Okano, K., Suzuki, Y., & Masaki, T. Over-the-scope clip system: A review of 1517 cases over 9 years. *J Gastroenterol Hepatol*. 2019;34(1):22-30. doi:10.1111/jgh.14402
65. Pines, G., Bar, I., Elami, A., Sapojnikov, S., Hikri, O., Ton, D., Mosenkis, B., & Melzer, E. Modified Endoscopic Vacuum Therapy for Nonhealing Esophageal Anastomotic Leak: Technique Description and Review of Literature. *J Laparoendosc Adv Surg Tech A*. 2018;28(1):33-40. doi:10.1089/lap.2017.0318
66. Guida AM, Leonetti G, Finizio R, Montagnese F, Efrati C, Sena G, Divizia A, Benavoli D (2022) Endoscopic solutions for colorectal anastomotic leaks. Techn Innov Gastrointest Endosc 24(1):57–65
67. Kouladouros K. Applications of endoscopic vacuum therapy in the lower gastrointestinal tract: Tips and tricks and a review of the literature. *Best Pract Res Clin Gastroenterol*. 2024;70:101927. doi:10.1016/j.bpg.2024.101927
68. Cereatti, F.; Grassia, R.; Drago, A.; Conti, C.B.; Donatelli, G. Endoscopic management of gastrointestinal leaks and fistulae: What option do we have? *World J. Gastroenterol.* **2020**, *26*, 4198–4217. https://doi.org/10.3748/wjg.v26.i29.4198.
